# Germination, root elongation, and photosynthetic performance of plants exposed to sodium lauryl ether sulfate (SLES): an emerging contaminant

**DOI:** 10.1007/s11356-021-12574-w

**Published:** 2021-02-01

**Authors:** Elisabetta Salvatori, Jasmin Rauseo, Luisa Patrolecco, Anna Barra Caracciolo, Francesca Spataro, Lina Fusaro, Fausto Manes

**Affiliations:** 1 Department of Environmental Biology, Sapienza University of Rome, Piazzale Aldo Moro, 5, 00185 Rome, Italy; 2 Present Address: ENEA, Italian National Agency for New Technologies, Energy and Sustainable Economic Development, SSPT-STS, R.C. Casaccia, Via Anguillarese, 301 - 00123 S.Maria di Galeria, Rome, Italy; 3 Institute of Polar Sciences - National Research Council (ISP-CNR), Via Salaria km 29.300, 00015 Monterotondo, Rome, Italy; 4 Water Research Institute - National Research Council (IRSA-CNR), Via Salaria km 29.300, 00015 Monterotondo, Rome, Italy

**Keywords:** Anionic surfactants, Plant stress, Chlorophyll fluorescence, JIP test, Gas exchanges, Microbial abundance, Microbial activity

## Abstract

The anionic surfactant SLES (sodium lauryl ether sulfate) is an emerging contaminant, being the main component of foaming agents that are increasingly used by the tunnel construction industry. To fill the gap of knowledge about the potential SLES toxicity on plants, acute and chronic effects were assessed under controlled conditions. The acute ecotoxicological test was performed on *Lepidum sativum* L. (cress) and *Zea mays* L. (maize). Germination of both species was not affected by SLES in soil, even at concentrations (1200 mg kg^−1^) more than twice higher than the maximum realistic values found in contaminated debris, thus confirming the low acute SLES toxicity on terrestrial plants. The root elongation of the more sensitive species (cress) was instead reduced at the highest SLES concentration. In the chronic phytotoxicity experiment, photosynthesis of maize was downregulated, and the photosynthetic performance (PI_TOT_) significantly reduced already under realistic exposures (360 mg kg^−1^), owing to the SLES ability to interfere with water and/or nutrients uptake by roots. However, such reduction was transient, likely due to the rapid biodegradation of the surfactant by the soil microbial community. Indeed, SLES amount decreased in soil more than 90% of the initial concentration in only 11 days. A significant reduction of the maximum photosynthetic capacity (Pn_max_) was still evident at the end of the experiment, suggesting the persistence of negative SLES effects on plant growth and productivity. Overall results, although confirming the low phytotoxicity and high biodegradability of SLES in natural soils, highlight the importance of considering both acute and nonlethal stress effects to evaluate the environmental compatibility of soil containing SLES residues.

**Supplementary Information:**

The online version contains supplementary material available at 10.1007/s11356-021-12574-w.

## Introduction

Sodium lauryl ether sulfate (SLES, molecular formula: CH_3_[CH_2_]_11_[OCH_2_CH_2_]_n_ OSO_3_Na^+^) is one of the most commonly used anionic surfactants for household and industrial applications. Due to its very low production cost and high emulsifying and foaming properties, it can be found in several products, such as detergents, cosmetics, and personal care products, in a concentration range varying from 0.1 to 50% (Barra Caracciolo et al. 2017). For these same properties, SLES is also the main component of numerous synthetic foaming agents used as lubricants for mechanized tunneling with the TBM-EPB (Tunnel Boring Machine-Earth Pressure Balance) technology (Baderna et al. 2015; Barra Caracciolo et al. 2017; Sebastiani et al. 2019). The soil debris produced by TBM-EPB are composed of a mixture of soil and rocks with the foaming agent used for soil conditioning, where SLES concentration can range from 40 to 500 mg kg^−1^ (Barra Caracciolo et al. 2017). Due to the ongoing and planned construction of tunnel infrastructures in Europe, the amount of spoil material deriving from soil excavations is continuously growing: in 2017, Italy alone produced 13.6 M t of soil debris (ISPRA 2019), and foaming agents containing SLES are reported to be used in several railway or highway tunneling construction sites (see, for example, Barra Caracciolo et al. 2019; Finizio et al. 2020; Mariani et al. 2020). The disposal of this large amount of soil debris can be an environmental issue for both the construction industry and the society, if they are not adequately managed.

Currently, neither EU (Directive 2008/98/CE) nor national legislations (e.g., Italian Decree D.P.R. 120/2017) set SLES concentration limits in soil debris (Barra Caracciolo et al. 2019), thus allowing the potential reuse of SLES-contaminated soils for different purposes in the environment, for example, as land filling material or in green areas. In this context, SLES contamination in soils represents an emerging problem, also posing a risk for water if its residues run off or leach from the destination site to water bodies in contact with it.

Recent studies have tested acute SLES toxicity on model organisms of water and soil communities (Baderna et al. 2015; Grenni et al. 2018; Galli et al. 2019; Finizio et al. 2020; Mariani et al. 2020), pointing out that, while SLES had severe effects on aquatic organisms, concentration levels usually found in spoil material did not exert toxic effects on soil animals, nor impaired acute ecotoxicological endpoints such as seed germination and root elongation of the tested plant species (*Cucumis sativus* L., *Sorghum saccharatum* L., and *Lepidium sativum* L.). It has been also shown that SLES is easily biodegraded in soils by natural microbial populations, with half-lives ranging from 8 to 46 days, depending on the soil type (Finizio et al. 2020; Pescatore et al. 2020). In the last years, some studies found bacterial consortia isolated from wastewater and activated sludge capable of degrading SLES. Most bacteria of the consortia identified belonged to Gammaproteobacteria, such as *Pseudomonas* and *Aeromonas* (Paulo et al. 2017), *Serratia*, *Enterobacter*, and *Alcaligenes* (Fedeila et al. 2018), and the *Azotobacter*, *Pseudomonas*, *Acinetobacter*, *Klebsiella*, and *Serratia* (Khleifat 2006). Moreover, a recent work identified a bacterial consortium isolated from a deep soil, capable of utilizing SLES as a sole carbon source and degrading it in few hours. The bacterial consortium was identified by the next generation sequencing (NGS) analysis and the main genera found were *Pseudomonas*, *Acinetobacter*, *Stenotrophomonas*, and *Pseudoxanthomonas*, confirming the predominant role of Gammaproteobacteria in SLES degradation (Rolando et al. 2020).

The acute ecotoxicological tests, using a single species in contact with different concentrations of a “pure” substance, are useful in ranking the relative toxicity of compounds vs others. Nevertheless, they do not take into consideration the overall biotic and abiotic factors that occur in a real matrix (e.g., soil), nor the whole range of possible stressful effects of chemicals on complex organisms. Indeed, several surfactants, if present in irrigation water or in soils, are known to exert detrimental effects on plants, particularly affecting ecophysiology and growth (Liwarska-Bizukojc 2009). For instance, Uzma et al. (2018) found that two detergents containing anionic surfactants, although not significantly affecting seed germination, were able to impair cell viability and light-harvesting pigments (chlorophyll a and b and carotenoids) in maize (*Zea mays* L.). Such negative effects on the photosynthetic apparatus have been also reported for bean plants (*Phaseoulus vulgaris* L.), treated with a domestic detergent containing anionic surfactants (Jovanić et al. 2010); as a consequence, a marked (− 45%) decrease in photosynthetic activity was observed. In addition, Masoudian et al. (2020) reported that an anionic surfactant (sodium dodecyl benzene sulfonate) triggered an oxidative stress response in the aquatic plant *Azolla filiculoides* Lam., reducing its chlorophyll content and growth rate.

To the best of our knowledge, no study has investigated the effects of SLES on plant ecophysiology and growth under nonlethal environmentally relevant concentrations found in soil excavation debris. Such effects should be taken into account in the risk assessment of this emerging pollutant, being critical for ecosystem protection. In this context, the present paper aims at (a) obtaining further evidences on the acute ecotoxicological effects of SLES on seed germination and root elongation of two plant species (*Lepidium sativum* L., cress, a reference species for phytotoxicity tests, and *Zea mays* L., maize, a “model species” in ecophysiological studies) and (b) investigating the potential nonlethal stress effects of SLES on the photosynthetic process of maize, also considering the role of the soil microbial community in SLES degradation. We hypothesized that environmentally relevant SLES concentrations are nonlethal to the considered plant species but are able to trigger a stress response and significantly impair the photosynthetic process.

## Materials and methods

The experiments were carried out in the Laboratory of Functional Ecology and Ecosystem Services at the Department of Environmental Biology, Sapienza University of Rome, Italy.

### Acute phytotoxicity tests on *Zea mays* and *Lepidium sativum*

Acute SLES phytotoxicity was evaluated by applying the germination test of Martignon (2009), based on the exposure of seeds of vascular plant species to an environmental matrix, or a chemical compound, for 72 ± 0.5 h in the dark at 25 ± 2 °C. The toxic effect on the reproductive (germination rate) and vegetative (root elongation) endpoints are then assessed. Commercially available seeds of *Zea mays* L. var. “Everta” (maize) and *Lepidium sativum* L. (cress) (Fratelli Ingegnoli, Milan, Italy) were used for the test. Cress is already a reference species for phytotoxicity tests (Baderna et al. 2015), while compatibility of maize seeds with the environmental growth conditions and time constraints of the method was verified before performing the test. Indeed, according to Martignon et al. (2009), and in agreement with the OECD guidelines for the testing of chemicals (OECD 2006), in order for a species to be considered suitable for the test, germination in the controls should be at least 70%. Since the germination rate of maize under such conditions was higher than 80%, it has been considered suitable for the test.

The conditioning agent SLES was purchased from BOC Sciences Inc., USA (CAS n. 68585-34-2, 70% purity). Three SLES concentrations were tested: 100, 300, and 1000 mg L^−1^. The stock SLES solution was prepared at 1000 mg L^−1^ in ultrapure water and then diluted to the final concentrations. SLES phytotoxicity was tested on both liquid and solid growth medium, using 100-mm Petri dishes, covered with 90-mm filter paper disks (Watman, Grade 1). The test in the liquid was performed by adding 5 mL of solution directly on the filter paper, on the top of which ten seeds were added. The control test was performed with 5 mL of ultrapure water. As for the test on the solid growth medium, 10 g of soil, obtained by mixing 50% garden soil (35% organic C, 11% humic C, 1.4% organic N, Compo Bio, Compo Agro Specialities Srl) and 50% sand (93% limestone, 5% quartz and flint, 1% sandstones and siltstones, 1% tuff), were plated in each dish, covered with filter paper, and then wetted at full capacity with 12 mL of SLES solution (12 mL of ultrapure water for controls), prior to adding 10 seeds per plate. The corresponding SLES concentrations of soil were therefore 120, 360, and 1200 mg kg^−1^, respectively: the first two amounts were in the range of those generally found in soil debris, while the latter one was more than twice higher than the maximum reported concentration (Barra Caracciolo et al. 2017). Each concentration was replicated in 4 plates for species and growth medium. All plates were sealed and placed at 25 ± 2 °C in a dark growth chamber. At the end of the incubation time (72 ± 0.5 h), the number of germinated seeds and the root elongation were assessed. The Germination Index (GI) was then calculated for each plate as:

1 $$ \mathrm{GI}=\mathrm{number}\ \mathrm{of}\ \mathrm{germinated}\ \mathrm{seeds}\times \mathrm{mean}\ \mathrm{root}\ \mathrm{length} $$

and then expressed as percentage per treatment (Martignon 2009; Baderna et al. 2015):

2 $$ \mathrm{GI}\%=\left(\mathrm{GI}\ \mathrm{average}\ \mathrm{treated}/\mathrm{GI}\ \mathrm{average}\ \mathrm{control}\right)\ast 100 $$

### Chronic phytotoxicity test on *Zea mays*

Maize has been chosen for the chronic phytotoxicity experiment due to its role as a “model species” in ecophysiological studies, as well as for its strategic and economic importance (Gulli et al. 2015).

#### Experimental design and plant growing conditions

Maize seeds were sown in 42 pots of 1.5 L each (4 seed per pot), filled with the same soil used for the acute phytotoxicity test, placed inside the “walk-in” chamber facility (2.5 m × 3.9 m × 3 m h) of the Department of Environmental Biology, Sapienza University of Rome (Salvatori et al. 2013). Inside the chamber, environmental parameters were maintained as follows: photosynthetic active radiation = 700 μmol m^−2^ s^−1^; photoperiod = 12 h; air temperature = 25.0 ± 2 °C; relative humidity = 60 ± 5%. During the whole experimental period, pots were randomly relocated in the chamber every day to prevent position effects. Starting from the 9th day after sowing (DAS), each pot was provided once a week with 30 mL of Hoagland’s No. 2 Basal Salt Mixture (Sigma-Aldrich Co) at ¼ of strength. At DAS 12, germinated plants were thinned to one per pot. At DAS 26, when plants had an average of 3.4 ± 0.5 leaves, the SLES treatment was applied. Pots were randomly divided into 3 experimental sets, of 14 pots each: C, control, not treated; T360 mg kg^−1^, treated with 360 mg kg^−1^ SLES; T1200 mg kg^−1^, treated with 1200 mg kg^−1^ SLES. These SLES concentrations were chosen on the basis of the results of the acute phytotoxicity test. SLES treatment was provided in 100 mL of deionized water per pot; concurrently, control plants were irrigated with 100 mL of deionized water.

#### Ecophysiological measurements

##### Gas exchange measurements

The Infra-Red Gas Analyzer CIRAS 2 (PP Systems, Amesbury, MA, USA) was used to measure net photosynthesis (Pn, μmolCO_2_ m^−2^ s^−1^), stomatal conductance (gs, mmolH_2_O m^−2^ s^−1^), leaf transpiration (E, mmolH_2_O m^−2^ s^−1^), and substomatal CO_2_ (Ci, ppm) at leaf level. Photosynthetically active radiation (PAR, μmol photons m^−2^ s^−1^), relative humidity (RH, %), air (Tamb, °C), and leaf (Tleaf, °C) temperatures were also recorded by the instrument. The ratio between substomatal (Ci) and ambient (Ca) CO_2_ concentration (Ci/Ca, dimensionless) was calculated.

Following Long and Bernacchi (2003) and Sharkey et al. (2007), the Pn/Ci response curves were also measured by using CIRAS 2, deriving the following parameters: in vivo apparent Rubisco activity (Vc_max_, mol m^−2^ s^−1^); CO_2_ compensation point (Γ, ppm), i.e., the point on the Pn/Ci response curve, where CO_2_ exchange from photosynthesis and that from respiration balance each other; and maximum net photosynthesis (Pn_max,_ μmolCO_2_ m^−2^ s^−1^) (Salvatori et al. 2013).

Steady-state gas exchanges were measured before the treatment at DAS 26, hereafter indicated as day of treatment (DOT) 0, and after 1, 2, 4, and 7 DOT, on the first fully expanded leaf at the top of each plant. Pn/Ci curves were measured during DOT 8, 9, and 10, on a total of 3 plants per treatment.

##### Chlorophyll “a” fluorescence and relative chlorophyll content measurements

Prompt chlorophyll fluorescence kinetic was measured in vivo by the HandyPea fluorimeter (Hansatech Instruments, Norfolk, UK), on the same leaves sampled for gas exchanges, dark adapted for 40 min by specific leaf clips (2 clips per leaves, positioned on the middle leaf blade). The JIP test parameters (Strasser et al. 2000) described in Table 1 were then derived (see Supplementary Material S1 for further details).

**Table 1:** Chlorophyll fluorescence parameters used in the text, calculated by the JIP test (Strasser et al. 2000)

JIP test parameters	
φ_Po_	Maximum quantum yield of photosystem II (PSII) primary photochemistry, measured on dark-adapted samples. Expresses the efficiency with which an absorbed photon will be trapped by the PSII reaction center (RC)
J-Phase or Ψ_Eo_	Probability that a trapped excitation by the PSII RC enters the electron transport chain
ABS/RC	Effective antenna size of an active RC. Expresses the total number of photons absorbed by chlorophyll molecules of all RC divided by the total number of active RCs
DI_0_/RC	Effective energy dissipation per RC
ΔV_I-P_	Amplitude of the IP-phase (Oukarroum et al. 2009). It indicates the efficiency of electron transport around the photosystem I (PSI) to reduce the final electron acceptors, i.e., ferredoxin and NADP (Ceppi et al. 2012)
PI_TOT_	Performance index (potential) for energy conservation from photons absorbed by PSII to the reduction of PSI end acceptors (Strasser et al. 2010)

See Supplementary Material S1 for further details

The relative chlorophyll content was measured by using a SPAD meter (Minolta), on the same leaves sampled for gas exchanges and chlorophyll fluorescence.

Both chlorophyll fluorescence and SPAD were measured during DOT 0, 1, 2, 4, 7, and 8.

#### Analytical and microbiological determinations

At DOT 11, all 45 pots (15 replicates for each condition: C, T360 mg kg^−1^ and T1200 mg kg^−1^) were collected destructively. Aliquots of both plants and soil were sampled from each pot condition and separately mixed for obtaining a composite sample. For the chemical and microbiological determinations, 5 sub-replicates were analyzed.

##### SLES analysis

All solvents used for chemical determinations were at HPLC grade and were obtained from VWR (Radnor, USA). SLES was extracted from fresh soil and lyophilized maize leaf samples by pressurized liquid extraction (PLE) using the Thermo Scientific Dionex ASE™ 150 (accelerated solvent extractor) system. Soil and leaves samples were mixed and homogenized with a dispersant agent (diatomaceous earth, Thermo 062819, USA) to fill the PLE cells. The operating PLE conditions are reported in Grenni et al. (2018) but using ultra-pure water (18 MΩ/cm quality, Millipore, Bedford, USA) as the extraction solvent.

SLES residual concentration in aqueous PLE extracts was determined following the optimized MBAS spectrophotometric method (Methylene Blue Active Substances) reported in Grenni et al. (2018). Briefly, SLES was extracted three times with chloroform to obtain a blue salt. The absorbance of the SLES-MBAS-chloroform complex was then measured using a UV/Vis spectrophotometer (650 nm Perkin Elmer 25). The calibration curves for lower (0.05–0.5 mg L^−1^) and higher (0.05–0.5 mg L^−1^) concentration ranges were acquired by the analysis of SLES standard solutions at different concentration levels. The limit of detection (LOD) was 0.013 mg L^−1^ (IUPAC 1999) and the PLE extraction recovery was 97.3 ± 0.6%.

The final extracts were also analyzed by LC-MS/MS (triple quadrupole mass spectrometer detector, equipped with an electrospray ionization detector, mod. API 3000, AB Sciex, Germany) in order to confirm the results obtained by the MBAS analyses. The injector valve (Rheodyne, mod. 7125) included a 20-μL loop. The chromatographic Luna column (250 × 4.6 mm, 5 μm C 18, Phenomenex, France), preceded by a guard column packed with the same stationary phase, was maintained at 25 °C. The isocratic elution was carried out at 1.0 mL/min flow rate, by using a mobile phase composed of ammonium acetate (30 mM water solution)/acetonitrile, 30:70 (v:v). The Analyst Service version 1.6 software allowed both instrument control and data acquisition. Further LC-MS/MS details are reported in Supplementary Material (S2).

SLES transportation from soil to maize leaves was evaluated by calculating the bioaccumulation factor (BAFs, where BAF plant-part = [SLES]leaves/[SLES]soil), according to Di Lenola et al. (2018).

#### Microbiological determinations in soil

##### Total microbial abundance and activity

Total microbial abundances of soil samples (no. of cells/g soil) were directly counted under a fluorescent microscope using the DNA fluorescent intercalant DAPI (4′,6-diamidino-2-phenylindole) as described in details in other works (Barra Caracciolo et al. 2019; Rauseo et al. 2019).

The overall activity of the microbial community was evaluated in soil samples with the dehydrogenase assay (DHA method). It is based on the reduction of the TTC (2,3,5-triphenyltetrazolium chloride) in TPF (triphenylformazan). Results are expressed as μg TPF/g soil. Details on this method are reported in Grenni et al. (2009).

#### Statistical analysis

Germination and ecophysiological data were analyzed by using Statistica 7.0 (StatSoft, Inc., Tulsa OK, USA). A two-way analysis of variance (ANOVA) was applied to the results of the germination test, by considering species and treatment as factors. The effects of SLES treatment on ecophysiological data at each measurement interval was tested by a one-way ANOVA. The significance of the differences among treatments was estimated by the post hoc Student–Newman–Keuls test at *p* ≤ 0.05. Time effects on physiological measurements were tested with repeated measures ANOVA, with treatment as between-subjects factor.

The statistical analysis of chemical data, microbial abundance, and dehydrogenase activity was performed using a one-way ANOVA, with significant differences at the *p* < 0.05 level. The PC Program used was SIGMASTAT. Both the chemical and the microbiological results performed at the end of the chronic phytotoxicity experiment are expressed as means ± standard errors (SE) of five values for each datum.

## Results

### Acute phytotoxicity: tests on *Zea mays* and *Lepidium sativum*

**Fig. 1:**
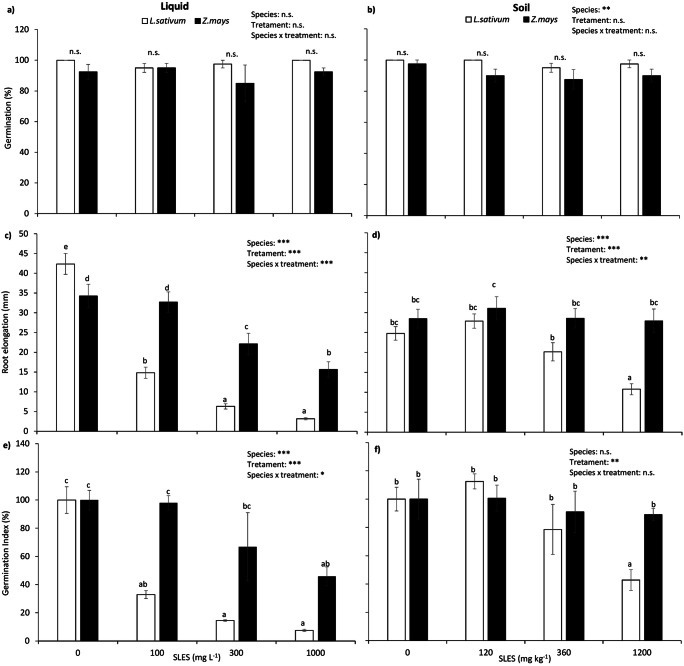
Germination rate (**a**, **b**), root elongation (**c**, **d**), and Germination Index (**e**, **f**), determined on cress (white bars) and maize (black bars) on liquid (**a**, **c**, **d**) and solid (**b**, **d**, **e**) growth medium. Data are shown as means ± standard error (*n* = 40). Different letters indicate statistically significant differences between means at *p* ≤ 0.05; insets show the results of the two-way ANOVA; asterisks showing the significance of factors/interaction (***p ≤ 0.001; **p ≤ 0.01; *p ≤ 0.05; n.s. = not significant, *p* > 0.05)

The acute phytotoxicity test highlighted that SLES treatment, even at the highest concentrations (1000 mg L^−1^ and 1200 mg kg^−1^), did not affect the germination rate of both cress and maize, either in liquid and solid growth medium (Fig. 1a and b). Root elongation was, instead, significantly affected, with differences between species and substrate (Fig. 1c and d). Cress resulted more sensitive than maize in both substrates: in the liquid, root elongation was significantly reduced in this species already at 100 mg L^−1^, while in maize a significant reduction was observed starting from the concentration of 300 mg L^−1^. At the highest concentration (1000 mg L^−1^), root elongation was reduced by 92.5% in cress and 54.3% in maize (Fig. 1c). The effect of SLES on root elongation was less marked in soil on both species, with cress showing significant reduction only at the highest concentration of 1200 mg kg^−1^ and maize showing no significant effect at all (Fig. 1d). The Germination Index showed the same pattern as root elongation, thus confirming the highest sensitivity to SLES of cress in respect to maize, and the lower toxic effect of the anionic surfactant in soil on both species: GI was reduced by 92.5% and 54.15% in cress and maize, respectively (Fig. 1e) in the liquid growth medium at the highest SLES concentration; in soil the reduction was 57% and 11%, respectively (Fig. 1f).

On the basis of these results, the SLES concentrations chosen for performing the chronic phytotoxicity test on *Zea mays* were 360 mg kg^−1^ (no evidence of acute effects) and 1200 mg kg^−1^ (significant acute effects on sensitive species only).

### Chronic phytotoxicity experiment on *Zea mays*

#### Steady-state gas exchanges

**Fig. 2:**
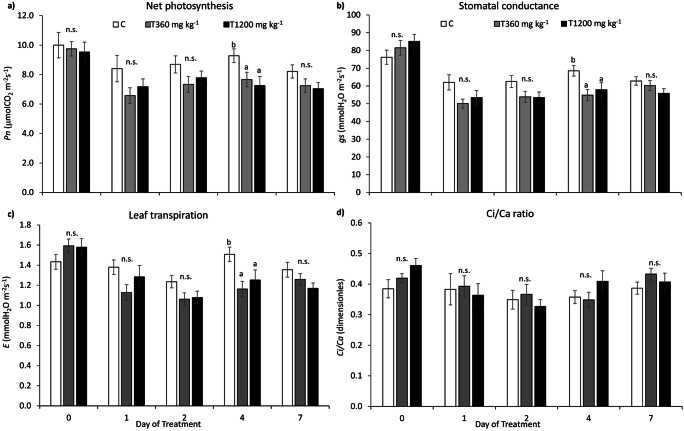
Net photosynthesis (Pn, **a**), stomatal conductance (gs, **b**), leaf transpiration (E, **c**), and the ratio between substomatal and external CO_2_ concentration (Ci/Ca, **d**) measured on maize plants. C = control, plants not treated with SLES; T360 mg kg^−1^ = plants treated with 360 mg kg^−1^ SLES; T1200 mg kg^−1^ = plants treated with 1200 mg kg^−1^ SLES. Data are shown as means ± standard error (*n* = 14). For each day of treatment, different letters indicate statistically significant differences between means at *p* ≤ 0.05 (n.s. = not significant, *p* > 0.05)

Net photosynthesis (Fig. 2a), stomatal conductance (Fig. 2b), and leaf transpiration (Fig. 2c) of maize plants were reduced in SLES-treated plants since DOT 1, but this effect resulted significant only at DOT 4, with no difference between the two SLES concentrations (Table 2a). The Ci/Ca ratio instead did not show significant differences between the experimental sets (Fig. 2d, Table 2a). Time effect was significant on all gas exchange parameters, and significant time * SLES interactions were evident on gs and E (Table 2b).

**Table 2:** Results (*p* values) of the ANOVA on gas exchanges (Pn, net photosynthesis; gs, stomatal conductance; E, leaf transpiration; Ci/Ca, ratio between substomatal and external CO_2_ concentration), JIP test parameters (φ_Po_, maximum quantum yield of PSII; Ψ_E0_, J-Phase; ABS/RC, energy absorption per RC; DI_0_/RC, energy dissipation per RC; ΔV_I-P_, amplitude of the I-P phase; PI_TOT_, total photosynthetic Performance Index); and relative chlorophyll content (SPAD) data

a) One-way ANOVA											
DOT	Pn	gs	E	Ci/Ca	φ_Po_	Ψ_Eo_	ABS/RC	DI_0_/RC	ΔV_I-P_	PI_TOT_	SPAD
0	0.895	0.293	0.254	0.088	0.224	0.528	0.052	0.082	0.956	0.513	0.259
1	0.167	0.071	0.141	0.887	0.658	0.540	0.954	0.887	0.310	0.759	0.535
2	0.189	0.092	0.108	0.627	**0.001**	0.600	**0.003**	**0.001**	**0.022**	**0.006**	0.535
4	**0.022**	**0.015**	**0.015**	0.256	**0.000**	0.949	**0.003**	**0.001**	0.449	**0.017**	0.471
7	0.143	0.173	0.124	0.371	**0.002**	0.660	**0.009**	**0.004**	0.194	**0.001**	**0.001**
8	*n.m.*	*n.m.*	*n.m.*	*n.m.*	0.196	0.144	0.064	0.091	0.170	0.086	*n.m.*
b) Repeated measurements ANOVA											
Factors	Pn	gs	E	Ci/Ca	φPo	ΨE_0_	ABS/RC	DI_0_/RC	ΔV_I-P_	PI_TOT_	SPAD
Time	**0.000**	**0.000**	**0.000**	**0.007**	**0.000**	**0.000**	**0.000**	**0.000**	**0.000**	**0.000**	**0.000**
Time * SLES	0.673	**0.031**	**0.021**	0.569	**0.001**	0.152	0.059	**0.007**	**0.005**	0.120	0.373

(a) One-way ANOVA between treatments, for each day of treatment (DOT), degree of freedom = 2; (b) Repeated measurements ANOVA on time and SLES effects, degree of freedom: time = 4, time * SLES = 8 for gas exchanges and relative chlorophyll content, degree of freedom: time = 5, time * SLES = 10 for JIP test parameters. Significant (*p* ≤ 0.05) factors are marked in bold (*n.m.* = not measured)

#### Chlorophyll “a” fluorescence and SPAD

**Fig. 3:**
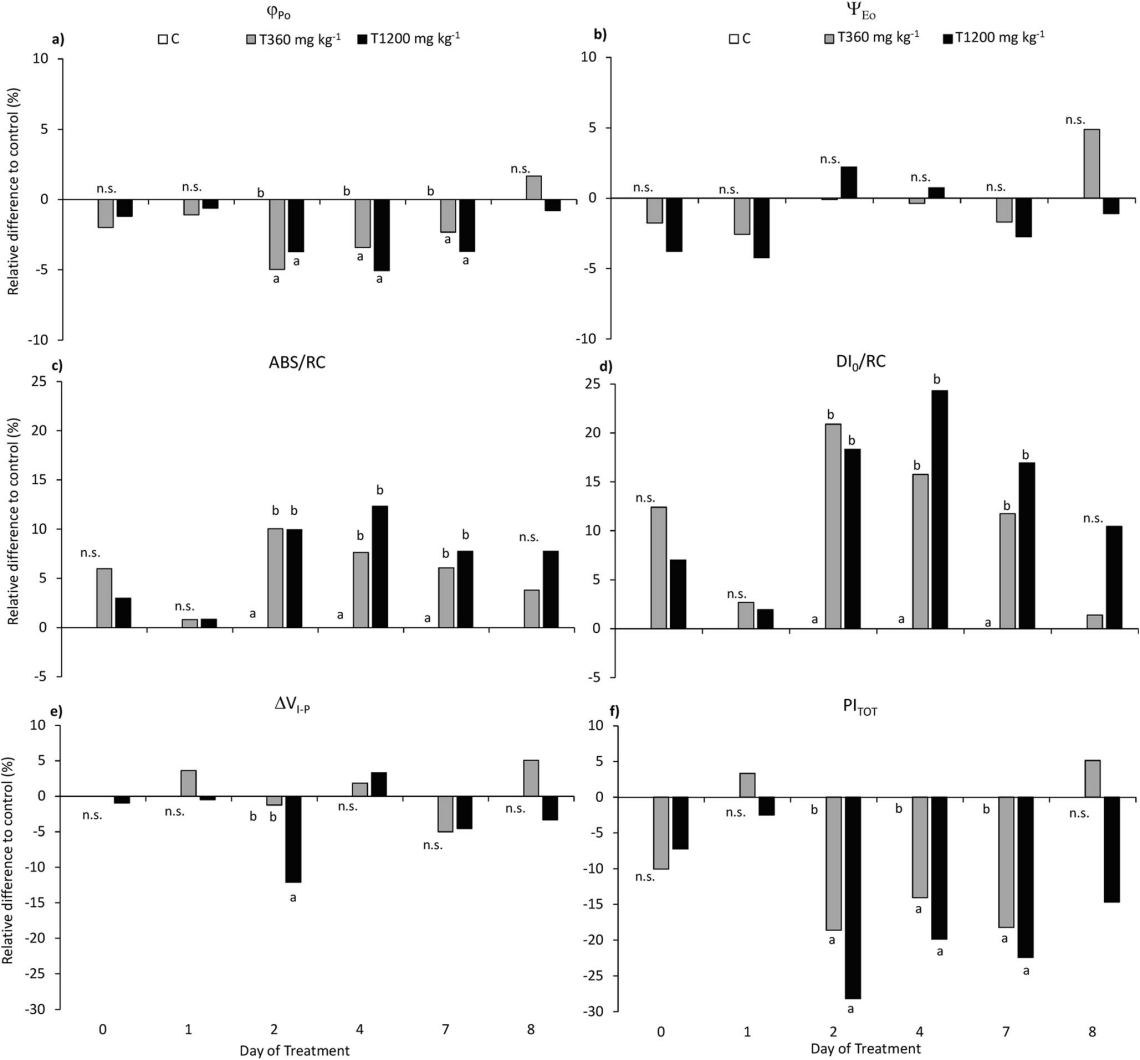
Maximum quantum yield of PSII (φ_Po_, **a**), J-Phase (Ψ_Εo_, **b**), energy absorption per RC (ABS/RC, **c**), energy dissipation per RC (DI_0_/RC, **d**), amplitude of the I-P phase (ΔV_I-P_, **e**), total photosynthetic Performance Index (PI_TOT_, **f**), measured on maize plants. C = control, plants not treated with SLES; T360 mg kg^−1^ = plants treated with 360 mg kg^−1^ SLES; T1200 mg kg^−1^ = plants treated with 1200 mg kg^−1^ SLES. Data are shown as percentage variation in respect to control values (*n* = 28). For each day of treatment, different letters indicate statistically significant differences between means at *p* ≤ 0.05 (n.s. = not significant, *p* > 0.05)

Figure 3 shows the trend of the selected JIP test parameters considered in this study, expressed as percentage variation in respect to control values. The maximum quantum yield of PSII (φ_Po_, Fig. 3a) showed slight but significant reductions on both SLES-treated sets starting from the second day of treatment, with a recovery at DOT 8 (Table 2a), while the J-Phase (Ψ_Εo_, Fig. 3b) was unaffected by SLES through the whole experiment (Table 2a). The specific energy fluxes, i.e., absorbance (ABS/RC, Fig. 3c) and dissipation (DI_0_/RC, Fig. 3d) per active reaction center, were significantly increased at both SLES levels from DOT 2 to 7 (Table 2a), while the I-P phase (ΔV_I-P_, Fig. 3e) showed a slight (− 12.1%) but significant reduction only for T1200 mg kg^−1^ plants at DOT2 (Table 2a). Finally, the total photosynthetic performance (PI_TOT_, Fig. 3f) was significantly reduced in both treatments from DOT 2 to 7 (Table 2a).

**Fig. 4:**
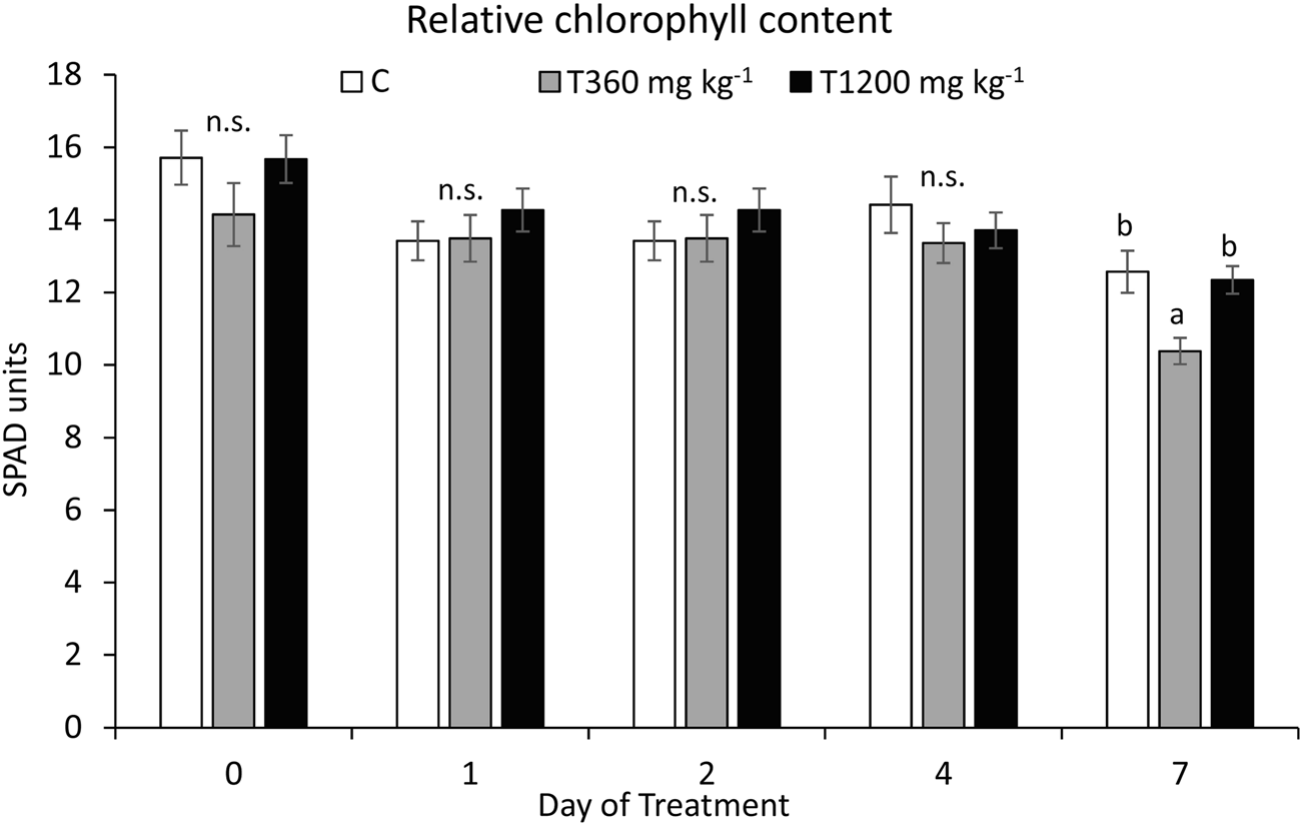
Relative chlorophyll content (SPAD units) measured on maize plants. C = control, plants not treated with SLES; T360 mg kg^−1^ = plants treated with 360 mg kg^−1^ SLES; T1200 mg kg^−1^ = plants treated with 1200 mg kg^−1^ SLES. Data are shown as means ± standard error (*n* = 28). For each day of treatment, different letters indicate statistically significant differences between means at *p* ≤ 0.05 (n.s. = not significant, *p* > 0.05)

The relative chlorophyll content (SPAD, Fig. 4) was unaffected by SLES treatment during the whole experiment. A slight significant reduction was evident only in T360 mg kg^−1^ at DOT 7 (Table 2a).

As observed for gas exchanges, time effect was significant for all chlorophyll fluorescence and SPAD parameters, while a significant time × SLES interaction was highlighted for φ_Po_, DI_0_/RC, and ΔV_I-P_ (Table 2b).

#### Pn vs Ci response curves

Table 3 shows the photosynthetic parameters derived from the Pn/Ci response curves, measured during DOT 8–10. No significant effect of SLES was evident on both the in vivo Rubisco carboxylation velocity (Vc_max_, mol m^−2^ s^−1^), and the CO_2_ compensation point (Γ, ppm), i.e., the point on the Pn/Ci response curve where CO_2_ exchange from photosynthesis and that from respiration balance each other. The maximum net photosynthesis (Pn_max_, μmolCO_2_ m^−2^ s^−1^) was instead significantly reduced (*p* = 0.000**)** by SLES, with no difference between the two treatment concentrations.

**Table 3:** Parameters derived from the Pn vs Ci response curves measured at the end of the experimental period

	Treatment						
	C		T360 mg kg^−1^		T1200 mg kg^−1^		
	Mean ± St. Err		Mean ± St. Err		Mean ± St. Err		*p* value
Vc_max_	0.09 ± 0.01	a	0.08 ± 0.01	a	0.08 ± 0.00	a	0.591
Γ	12.47 ± 4.82	a	7.27 ± 2.78	a	10.72 ± 1.08	a	0.517
Pn_max_	14.50 ± 0.37	a	9.37 ± 0.34	b	10.22 ± 0.26	b	**0.000**

Vc_max_ (mol m^−2^ s^−1^) = in vivo apparent Rubisco activity; Γ (ppm) = CO_2_ compensation point; Pn_max_ (μmol m^−2^ s^−1^) = maximum rate of net photosynthesis. Data are shown as means ± standard error (*n* = 3); for each parameter, different letters indicate statistically significant differences between treatments at *p* ≤ 0.05 (post hoc test). The *p* values of the one-way ANOVA are also reported (significant *p* ≤ 0.05 differences marked in bold); degree of freedom = 2

#### Anionic surfactant concentration in soil and maize leaves

**Fig. 5:**
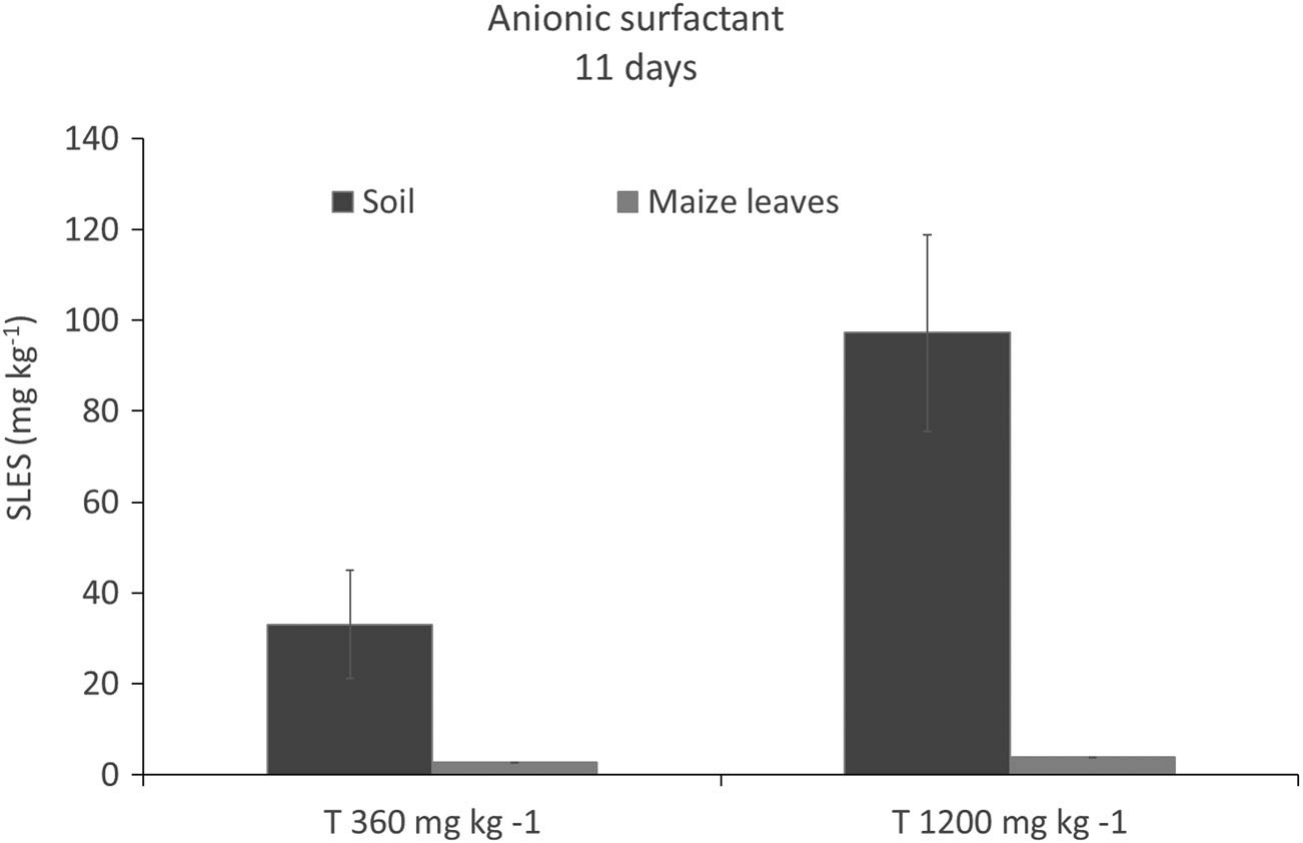
Residual concentrations of SLES (mg kg^−1^) measured in soil and maize leaves at the end of the chronic phytotoxicity experiment (DOT 11). Data are shown as means ± standard error (*n* = 5). T360 mg kg^−1^ SLES initial concentration; T1200 mg kg^−1^ SLES initial concentration

The average SLES concentrations detected at DOT 11 in the T360 mg kg^−1^ and T1200 mg kg^−1^ conditions, both in soil and leaves, are shown in Fig. 5.

SLES decreased significantly both in T360 mg kg^−1^ and T1200 mg kg^−1^ conditions, with a reduction of 90% from its initial concentrations; in fact, the final amounts were 33.1 ± 8.9 and 97.3 ± 11.6 mg kg^−1^, respectively (Fig. 5).

A SLES residual concentration of 2.7 ± 0.02 and 3.9 ± 0.08 mg kg^−1^ was observed in maize leaves in T360 mg kg^−1^ and T1200 mg kg^−1^, respectively. Finally, the leaf BAF, calculated at DOT 11, was 0.08 and 0.04 for T360 mg kg^−1^ and T1200 mg kg^−1^, respectively. Both values were < 1, suggesting that maize was not able to bioaccumulate SLES in leaves.

#### Soil microbial abundance and activity

The total microbial abundance (no. of cells/g soil) and activity (μg TPF/g soil) in SLES-treated (T360 mg kg^−1^ and T1200 mg kg^−1^) and control (C) soils measured at the end (11 days) of the chronic phytotoxicity experiment (DOT 11) are reported in Table 4.

**Table 4:** Total microbial abundance (no. of cells/g dry soil) and microbial activity (dehydrogenase, μg TPF/g dry soil) at day 11 in control, T360 mg kg^−1^, and T1200 mg kg^−1^ conditions

Treatment			
	C	T 360 mg kg^−1^	T 1200 mg kg^−1^
Total microbial abundance (no. of cells/g)	6.48E + 07 ± 1.2E + 07^a^	4.01E + 07 ± 1.8E + 06^b^	7.19E + 07 ± 3.6E + 06^a^
Microbial activity (μg TPF/g)	93.76 ± 41.74^a^	69.14 ± 5.43^a^	88.13 ± 5.49^a^

Each value is the average of 5 replicates ± standard error and for each parameter, different letters indicate statistically significant differences between treatments at *p* ≤ 0.05

The microbial abundance (no. of cells/g dry soil) in the T360 mg kg^−1^ condition was significantly lower (*p* < 0.05) than in the control one (Table 4). However, this parameter was not significantly different between the soil initially treated with SLES at a higher concentration (T1200 mg kg^−1^) and the control. The comparison between SLES-treated and control samples did not indicate significant differences in terms of microbial activity.

## Discussion

The overall results of the germination test confirmed the low ecotoxicity of SLES on the considered plants (*Lepidium sativum* and *Zea mays*), already highlighted in previous studies (Baderna et al. 2015; Grenni et al. 2018; Galli et al. 2019; Finizio et al. 2020). In particular, the germination process, i.e., the reproductive endpoint, appears to be unaffected by SLES, in both species and growth media. Indeed, Baderna et al. (2015) reported a No-Observed-Adverse-Effect-Concentration (NOAEC) of 1.5 to 3.8 g kg^−1^ of soil for three foaming agents containing a maximum 30% of SLES, a value that is well above our highest treatment concentration of 1200 mg kg^−1^, as well as above the realistic contamination levels of excavated soils (40–500 mg kg^−1^, Barra Caracciolo et al. 2017). On the contrary, the vegetative endpoint (root elongation) was significantly affected by SLES in both species, with a higher sensitivity of cress than maize, thus suggesting the possibility of potential negative SLES effects on plant growth. Anionic surfactants are known to primary impact the cell membranes, by either inserting into membrane phospholipids, or denaturizing and binding to the cell wall proteins, thus changing membrane permeability and eventually causing membrane disruption (Cserhati et al. 2002). In plant roots, this may result in altered membrane-dependent processes such as water and nutrient uptake, which ultimately affect plant growth. Besides, surfactants can change the physicochemical properties of growth medium, for example, increasing its osmotic potential, further affecting the water uptake process by roots (Wiel-Shafran et al. 2006; Heidari 2012). The observed effects on root elongation were substrate-dependent: in soil, the toxicity of SLES was reduced, possibly due to the adsorption of the surfactant to soil organic matter that reduces its availability for plant uptake (Finizio et al. 2020). Interestingly, the observed lack of acute SLES effects on both cress and maize for concentration values as high as 360 mg kg^−1^ in soil is in agreement with the work of Finizio et al. (2020), who reported a 50% inhibition of growth of cress seedlings starting at 433 mg kg^−1^ SLES.

The chronic phytotoxicity experiment confirmed SLES as a degradable compound in soil, with a decrease of 90% of its initial concentrations (360 and 1200 mg kg^−1^) in only 11 days in plant presence. This disappearance time observed was faster than that found in other works with lower initial SLES concentrations (70–100 mg kg^−1^) and where 90% of degradation was observed after 21 days (Barra Caracciolo et al. 2019). Other experiments reported the SLES biodegradability in soil, with half-lives ranging from 8 to 46 days, depending on the site-specific conditions such as soil lithological characteristics, soil depth, carbon content, the initial anionic surfactant concentration, and, above all, the abundance of soil microorganisms (Barra Caracciolo et al. 2019; Finizio et al. 2020). In this chronic phytotoxicity experiment, the microbial community showed much higher values of both microbial abundance (no. of cells/g dry soil 5E + 07) and activity (mean μg TPF/g dry soil 80) than those found in other works (Barra Caracciolo et al. 2019; Patrolecco et al. 2020). SLES biodegradation is possible thanks to several bacterial species such as *Citrobacter braakii* (Dhouib et al. 2003), *Aeromonas hydrophila*, *Pseudomonas stutzeri*, *Pseudomonas nitroreducens* (Paulo et al. 2017), *Alcaligenes faecalis*, *Enterobacter cloacae*, and *Serratia marcescens* strains (Fedeila et al. 2018) commonly found in various environments (Rolando et al. 2020). A possible degradation pathway for SLES is reported to be an initial ether cleavage and the sulfate ion as the final released product (Hales et al. 1986; Budnik et al. 2016). It is well known that sulfur has a key role both in plant-feeding and in the synthesis of the sulfur-containing amino acids (such as cysteine and methionine), as well as of other compounds playing important physiological functions (e.g., glutathione or ferredoxin) (Kowalska 2004). Consequently, the prompt degradation found in this experiment can also be ascribable to the positive interactions between plant roots and the soil microbial community. In fact, it is known that plant presence affects positively the microbial abundance and dehydrogenase activity (Barra Caracciolo et al. 2015; Di Lenola et al. 2018). For example, the organic substances released from roots may support higher microbial biomass and activity than those in the bulk soil. Indeed, the rhizosphere is a microhabitat where the microbial community interactions with plant species can also improve the biodegradation of contaminants in the so called “plant-assisted bioremediation” (Wenzel 2009; Ancona et al. 2017). However, further experiments are necessary for distinguishing the role of microorganisms and plants in SLES removal.

Regarding plant physiological response, although the SLES effect on steady-state gas exchanges was significant at DOT4 only, the JIP test revealed that a downregulation of the PSII photochemistry was occurring since the 2nd day of treatment. This mechanism involved a reduction of the maximum photosynthetic quantum yield (φ_Po_) and an increase of the specific energy fluxes ABS/RC and DI_0_/RC, related to a decrease in active RCs that are converted into “silent centers” (i.e., RCs that thermally dissipate the excess trapped energy (Strasser et al. 2004). Consequently, the plant photosynthetic performance is reduced, as highlighted by the significant decrease of PI_TOT_ in both T1200 mg kg^−1^ and T360 mg kg^−1^. This represents a conservative photoprotective strategy (Bussotti et al. 2020), which prevents irreversible injuries to the photosynthetic apparatus when photosynthesis is limited by environmental stress factors, as described in plants exposed to drought (Oukarroum et al. 2007; Strasser et al. 2010; Salvatori et al. 2016), salt stress (Fusaro et al. 2014; Kalaji et al. 2018), heavy metals (Bernardini et al. 2016), or tropospheric ozone (Bussotti et al. 2011; Salvatori et al. 2013, 2015; Fusaro et al. 2017). In our case, the observed PSII downregulation was likely triggered by the capacity of SLES to interfere with water and/or nutrients uptake by roots, in agreement with what is observed for root elongation in the germination test. Indeed, the chemical analysis of leaf tissue showed a low concentration of SLES in the leaf biomass and BAF values close to 0 for both T1200 mg kg^−1^ and T360 mg kg^−1^, suggesting a negligible transport of SLES into maize leaves, thus supporting the hypothesis of a surfactant effect mainly exerted at the root level. In addition, the rapid degradation of SLES not only allowed the recovery of the overall photosynthetic performance already at DOT8 but likely played a role in preventing the occurrence of more severe detrimental effects on photosynthesis: the J-Phase (Ψ_Eo_) and the chlorophyll content were, in fact, unaffected, while the functionality of PSI (ΔV_I-P_, Ceppi et al. 2012), which is frequently altered under oxidative stress conditions (Salvatori et al. 2014), showed a transient decrease at DOT 2 only at the highest SLES concentration of 1200 mg kg^−1^. It is, however, worth to underline that a significant reduction of the maximum photosynthetic capacity (Pn_max_) was highlighted by the Pn/Ci curves at the end of the experiment, thus suggesting a possible persistence of detrimental SLES effects on plant growth and productivity.

## Conclusions

Our initial hypothesis was verified, showing that (i) the germination process of cress and maize is not affected by SLES in soil, but the highest SLES concentration exerts a detrimental effect on root elongation of the more sensitive species (cress); (ii) the photosynthetic performance of maize is negatively affected by SLES already under realistic soil exposure levels; such effect is, however, transient and disappears after 8 days, likely due to the rapid biodegradation of the surfactant by the soil microbial community.

Our study, although confirming the low phytotoxicity and high biodegradability of SLES in natural soils, shows that this pollutant exerts non-negligible stress effects on plant photosynthetic performance, thus pointing out the importance of evaluating both acute and nonlethal effects when considering the possible environmental reuse of soil debris from excavation works. Starting from these results, further experiments should test nonlethal SLES effects on plant species differing in their sensitivity to this surfactant, and in different soil types, evaluating also the concurrent SLES biodegradability. Finally, the role of plants and their interaction with the microbial community in accelerating SLES biodegradation deserve further investigations, also in view of possible exploitation for “plant-assisted bioremediation” of SLES-contaminated debris, before their reuse in the environment.

## Supplementary information

ESM 1 (DOCX 21 kb)

## Data Availability

All data are available from the authors upon request to the corresponding author.

## Abbreviations

ABS/RC: effective antenna size of an active reaction center
BAF: bioaccumulation factor
Ca: ambient CO_2_ concentration
Ci: substomatal CO_2_ concentration
Ci/Ca: ratio between substomatal and ambient CO_2_
DAS: days after sowing
DI_0_/RC: energy dissipation per active reaction center
DOT: day of treatment
E: leaf transpiration
GI: Germination Index
gs: stomatal conductance
PI_TOT_: total photosynthetic performance index
PLE: pressurized liquid extraction
Pn: net photosynthesis
Pn_max_: maximum net photosynthesis at saturating light and CO_2_ concentration
PSI: photosystem I
PSII: photosystem II
RC: reaction center
RH: relative air humidity
SLES: sodium lauryl ether sulfate
Tamb: ambient temperature
Tleaf: leaf temperature
Vc_max_: in vivo apparent Rubisco activity
Γ: CO_2_ compensation point
Ψ_Eo_: electron transport probability
ΔV_I-P_: amplitude of the I-P phase of the fluorescence induction curve
φ_Po_: maximum quantum yield of photosystem II (PSII) primary photochemistry
