# Correction to: Germination, root elongation, and photosynthetic performance of plants exposed to sodium lauryl ether sulfate (SLES): An emerging contaminant

**DOI:** 10.1007/s11356-023-29404-w

**Published:** 2023-08-22

**Authors:** Elisabetta Salvatori, Jasmin Rauseo, Luisa Patrolecco, Anna Barra Caracciolo, Francesca Spataro, Lina Fusaro, Fausto Manes

**Affiliations:** 1 Department of Environmental Biology, Sapienza University of Rome, Piazzale Aldo Moro, 5, 00185 Rome, Italy; 2 Present Address: ENEA, Italian National Agency for New Technologies, Energy and Sustainable Economic Development, SSPT-STS, R.C. Casaccia, Via Anguillarese, 301 - 00123 S. Maria di Galeria, Rome, Italy; 3 Institute of Polar Sciences - National Research Council (ISP-CNR), Via Salaria km 29.300, 00015 Monterotondo, Rome, Italy; 4 Water Research Institute - National Research Council (IRSA-CNR), Via Salaria km 29.300, 00015 Monterotondo, Rome, Italy; 5 Institute of BioEconomy - National Research Council (IBE-CNR), Via dei Taurini 19, 00185 Rome, Italy

**Correction to: Environmental Science and Pollution Research (2021) 28:27900–27913**

10.1007/s11356-021-12574-w

The correct affiliation of Lina Fusaro is shown in this paper.

