# Supplementary material for: Germination, root elongation, and photosynthetic performance of plants exposed to sodium lauryl ether sulfate (SLES): an emerging contaminant
Source: Environ Sci Pollut Res Int. 2021 Feb 1;28(22):27900–13. doi: 10.1007/s11356-021-12574-w (PMC8164587; doi:10.1007/s11356-021-12574-w)
Supplement: Supplementary file 1 — (DOCX 21 kb) [file 11356_2021_12574_MOESM1_ESM.docx]

**Supplementary material**

**S1: Details of the JIP-Test analysis on chlorophyll fluorescence measurements**

The measured polyphasic prompt fluorescence transient (FT), plotted on a logarithmic time scale, exhibits a series of steps labeled as O (F_0_, when all the reaction centers, RCs, of the PSII are open) J (2 ms), I (30 ms) and maximum P level (F_m_, when all the Photosystem II, PSII, reaction centers are fully reduced). The O–J part of the FT is called “single turnover region” and expresses the photochemical events, related to the accumulation of reduced Q_A_ (primary electron acceptor quinone of PSII). The J–I–P region is called “multiple turnover region” and reflects the velocity of ferredoxine reduction beyond Photosystem I (PSI). The JIP-test analysis (Strasser et al. 2000) was applied to the FT by the Biolyzer software (Bioenergetics Lab., Geneva, CH), thus deriving the following parameters: from the single turnover region, the maximum quantum yield of PSII primary photochemistry measured on dark-adapted samples (ϕ_Po_), expressing the probability that an absorbed photon will be trapped by the PSII reaction centre; JPhase or Ψ_Eo_, expressing the probability that the energy of a trapped excitation is used for electron transport beyond QA; the energy absorption (ABS/RC) and dissipation (DI_0_/RC) per RC. From the multiple turnover region, the relative amplitude of the I-P phase of the fluorescence transient ΔV_I-P_ (Oukarroum et al. 2009), indicating the efficiency of electron transport around the PSI to reduce the end acceptors beyond PSI (Ceppi et al. 2012), was calculated. The multiparametric Photosynthetic Performance Index total (PI_TOT_), that synthesizes the potential for energy conservation from photons absorbed by PSII to the reduction of PSI end acceptors, was also calculated as PI_TOT_ = (RC/ABS)[ϕ_Po_/(1- ϕ _Po_)][Ψ_Eo_/(1-Ψ_Eo_)][δ_Ro_/(1-δ_Ro_)], where δ_Ro_ represents the probability that an electron is transported from the reduced plastoquinon to the electron acceptor side of PSI (Strasser et al. 2010).

**S2: SLES analysis, LC-MS/MS details**

In order to optimize the MS/MS conditions, SLES standard (10 mg L^-1^), containing the mobile phase, was firstly infused into the MS/MS through a syringe pump (flow rate of 10 µL/min) The instrument was operated in the positive ion mode (ESI+). The ionspray voltage was +5 kV, while nebulizer and curtain gas were at 8 and 8 units, respectively. Nitrogen was used as both collision and drying gas. Declustering, focusing and entrance potential were set at 100.9, 110 and 10.8 V, respectively. Full scan spectra were acquired in the mass range of m/z 150–900.When SLES solutions were infused, the most abundant mass recognized had m/z of 416.2, even if the m/z 512.3 and 556.4 occurred with a relevant signal. All these masses are related to adducts formed by SLES with ammonium acetate. In particular the m/z 416.2 is associated with the SLES-ammonium adduct ([M+NH_4_]^+^ with 3 ethoxylated (EO)groups; C12-3EO), while 512.2 m/z and 556.4 m/z correspond to two different desulfated ammonium adduct ([M+NH_4_-SO_3_]^+^). These results are also confirmed by Massey et al. (2010), reporting the same adducts for SLES. Multiple reaction monitoring (MRM) detection was based on the precursor (416.2 m/z) and product ion transitions (89.0 and 133.0 m/z) and on the comparison of the retention time (RT, 7.1 min.).
